# Supplementary material for: Combined Application of Pan-AKT Inhibitor MK-2206 and BCL-2 Antagonist Venetoclax in B-Cell Precursor Acute Lymphoblastic Leukemia
Source: Int J Mol Sci. 2021 Mar 9;22(5):2771. doi: 10.3390/ijms22052771 (PMC7967241; doi:10.3390/ijms22052771)
Supplement: Supplementary file 1 [file ijms-22-02771-s001.pdf]

## Supplementary materials

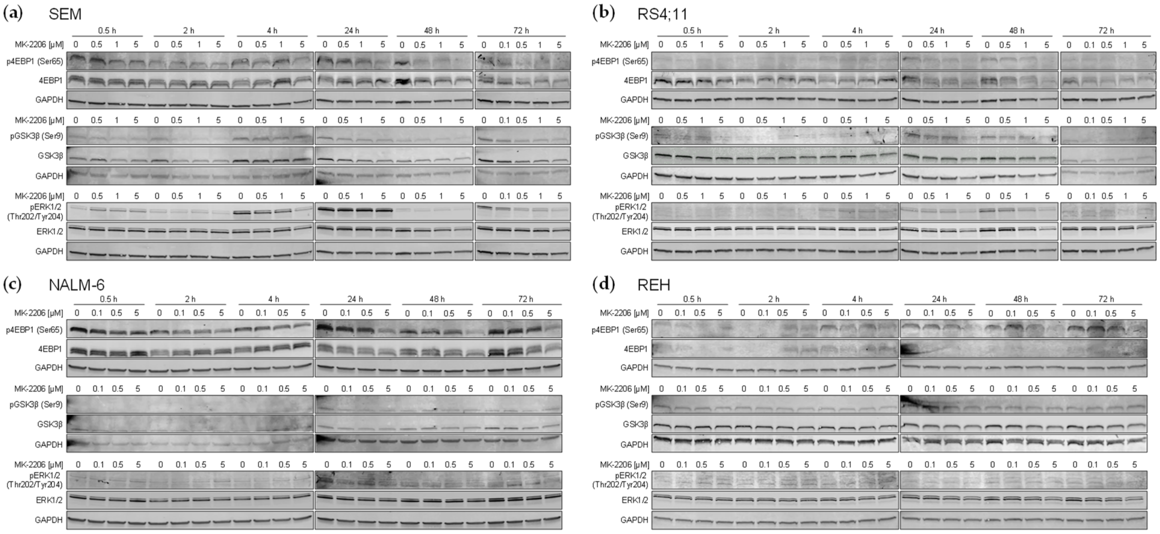


**Figure S1.** Analysis of phosphorylated and total 4EBP1, GSK3β and ERK1/2 protein expression by immunoblotting in SEM (**a**), RS4;11 (**b**), NALM-6 (**c**) and REH (**d**). Cells were incubated with the stated concentrations of MK-2206 for the indicated time periods. The displayed immunoblots are representative captions of at least two individual biological replicates. GAPDH was used as internal loading control. Blots were processed and cropped using Image Studio Lite 5.2 software and MS PowerPoint (2011) to improve clarity and conciseness.

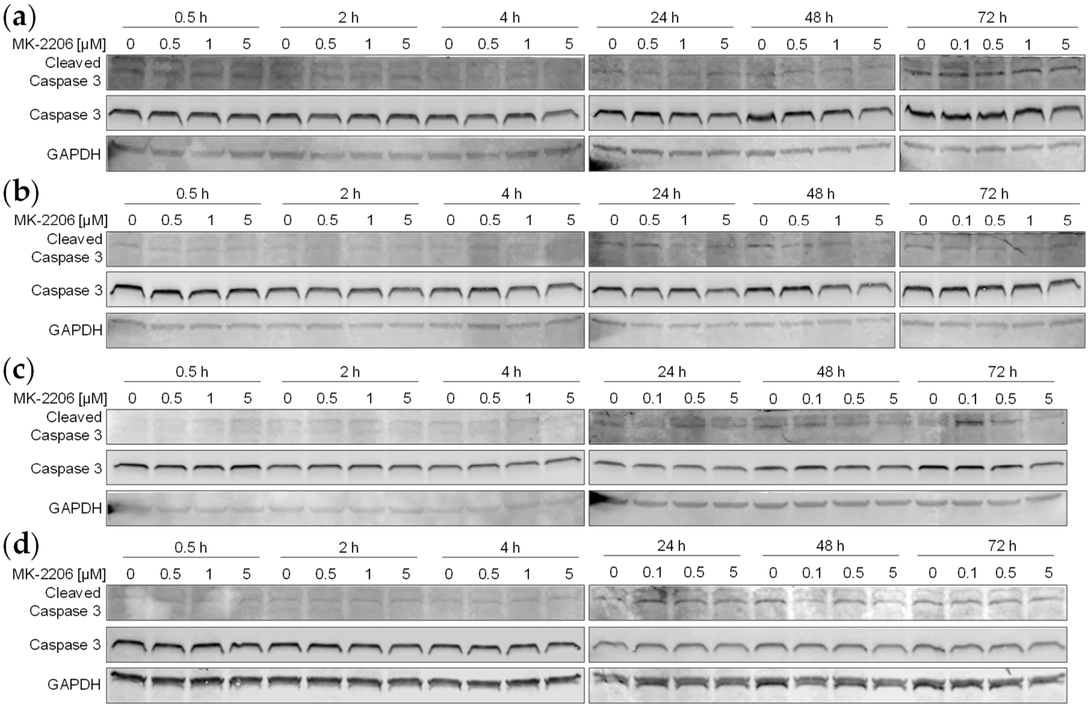


**Figure S2.** Analysis of apoptosis induction by cleaved and total Caspase 3 expression via immunoblotting in SEM (**a**), RS4;11 (**b**), NALM-6 (**c**) and REH (**d**). Cells were incubated with the stated concentrations of MK-2206 for the indicated time periods. GAPDH was used as internal loading control. Blots were processed and cropped using Image Studio Lite 5.2 software and MS PowerPoint (2011) to improve clarity and conciseness.

**Table S1.** *P* values of SEM cells treated with increasing concentrations of MK-2206 compared to DMSO-treated control cells. Three to five individual biological replicates were considered for the statistical calculation of significance by student's t test after testing Gaussian distribution. Significance is defined by $P < 0.05$ (*), $P < 0.01$ (**) and $P < 0.001$ (***).

| | Proliferation 48 h | Proliferation 72 h | Metabolic activity 48 h | Metabolic activity 72 h |
|---|---|---|---|---|
| **0.01 µM** | 0.1110 | 0.2396 | 0.0448 * | 0.5826 |
| **0.05 µM** | 0.0149 * | 0.1865 | 0.0446 * | 0.5267 |
| **0.1 µM** | 0.0015 ** | 0.1226 | 0.0141 * | 0.0362 * |
| **0.5 µM** | 0.0006 *** | 0.0002 *** | 0.0003 *** | 0.0012 ** |
| **1.0 µM** | 0.0009 *** | 0.0001 *** | 0.0004 *** | 0.0004 *** |
| **5.0 µM** | 0.0002 *** | 0.0000 *** | 0.0001 *** | 0.0000 *** |
| **10.0 µM** | 0.0002 *** | 0.0001 *** | 0.0023 *** | 0.0001 *** |

**Table S2.** *P* values of SEM cells treated with increasing concentrations of MK-2206 compared to DMSO-treated control cells. Three to five individual biological replicates were considered for the statistical calculation of significance by student's t test after testing Gaussian distribution. Significance is defined by $P < 0.05$ (*), $P < 0.01$ (**) and $P < 0.001$ (***).

| | Proliferation 48 h | Proliferation 72 h | Metabolic activity 48 h | Metabolic activity 72 h |
|---|---|---|---|---|
| **0.01 µM** | 0.3819 | 0.7044 | 0.0264 * | 0.7703 |
| **0.05 µM** | 0.6704 | 0.9160 | 0.0118 * | 0.4330 |
| **0.1 µM** | 0.2955 | 0.0338 * | 0.0136 * | 0.1119 |
| **0.5 µM** | 0.0311 * | 0.0100 * | 0.0010 ** | 0.0012 ** |
| **1.0 µM** | 0.0101 * | 0.0275 * | 0.0008 *** | 0.0002 *** |
| **5.0 µM** | 0.0006 *** | 0.0036 ** | 0.0000 *** | 0.0000 *** |
| **10.0 µM** | 0.0025 ** | 0.0008 *** | 0.0003 *** | 0.0000 *** |

**Table S3.** *P* values of SEM cells treated with increasing concentrations of MK-2206 compared to DMSO-treated control cells. Three to five individual biological replicates were considered for the statistical calculation of significance by student's t test after testing Gaussian distribution. Significance is defined by $P < 0.05$ (*), $P < 0.01$ (**) and $P < 0.001$ (***).

| | Proliferation 48 h | Proliferation 72 h | Metabolic activity 48 h | Metabolic activity 72 h |
|---|---|---|---|---|
| **0.01 µM** | 0.4594 | 0.4237 | 0.1305 | 0.6046 |
| **0.05 µM** | 0.0464 * | 0.1359 | 0.1320 | 0.8551 |
| **0.1 µM** | 0.0208 * | 0.0094 ** | 0.0346 * | 0.9930 |
| **0.5 µM** | 0.0126 * | 0.0002 *** | 0.0040 ** | 0.0038 ** |
| **1.0 µM** | 0.0046 ** | 0.0015 ** | 0.0030 ** | 0.0030 ** |
| **5.0 µM** | 0.0003 *** | 0.0000 *** | 0.0002 *** | 0.0005 *** |
| **10.0 µM** | 0.0000 *** | 0.0000 *** | 0.0000 *** | 0.0000 *** |

**Table S4.** *P* values of SEM cells treated with increasing concentrations of MK-2206 compared to DMSO-treated control cells. Three to five individual biological replicates were considered for the statistical calculation of significance by student's t test after testing Gaussian distribution. Significance is defined by $P < 0.05$ (*), $P < 0.01$ (**) and $P < 0.001$ (***).

| | Proliferation 48 h | Proliferation 72 h | Metabolic activity 48 h | Metabolic activity 72 h |
|---|---|---|---|---|
| **0.01 µM** | 0.5476 | 0.0302 * | 0.6741 | 0.5963 |
| **0.05 µM** | 0.4013 | 0.0905 | 0.0607 | 0.0162 * |
| **0.1 µM** | 0.0998 | 0.0274 * | 0.0026 ** | 0.0023 ** |
| **0.5 µM** | 0.0022 ** | 0.0061 ** | 0.0014 ** | 0.0009 *** |
| **1.0 µM** | 0.0412 * | 0.0043 ** | 0.0015 ** | 0.0035 ** |
| **5.0 µM** | 0.0050 ** | 0.0060 ** | 0.0008 *** | 0.0015 ** |
| **10.0 µM** | 0.0001 *** | 0.0001 *** | 0.0001 *** | 0.0000 *** |

**Table 5.** List of antibodies used for immunoblot analyses.

| Antibody | Host | Clone | Dilution | Supplier |
|---|---|---|---|---|
| GAPDH | Mouse | ZG003 | 1:20,000 | Invitrogen |
| Phospho AKT (Ser473) | Rabbit | polyclonal | 1:500 | Cell Signaling |
| AKT | Rabbit | polyclonal | 1:1,000 | Cell Signaling |
| Phospho 4EBP1 (Ser65) | Rabbit | 174A9 | 1:1,000 | Cell Signaling |
| 4EBP1 | Rabbit | polyclonal | 1:1,000 | Cell Signaling |
| Phospho GSK3β (Ser9) | Mouse | D2Y9Y | 1:500 | Cell Signaling |
| GSK3β | Mouse | 3D10 | 1:1,000 | Cell Signaling |
| Phospho-ERK (Tyr202/Tyr204) | Rabbit | polyclonal | 1:1,000 | Cell Signaling |
| ERK | Rabbit | 137F5 | 1:1,000 | Cell Signaling |
| Cleaved Caspase-3 (Asp175) | Rabbit | D175 | 1:500 | Cell Signaling |
| Caspase-3 | Rabbit | polyclonal | 1:500 | Cell Signaling |
| IRDye 680 goat anti-mouse | Goat | | 1:20,000 | LI-COR |
| IRDye 800 goat anti-mouse | Goat | | 1:20,000 | LI-COR |
| IRDye 680 goat anti-rabbit | Goat | | 1:20,000 | LI-COR |
| IRDye 800 goat anti-rabbit | Goat | | 1:20,000 | LI-COR |
